# Drought Tolerance of Soybean (*Glycine max* L. Merr.) by Improved Photosynthetic Characteristics and an Efficient Antioxidant Enzyme Activities Under a Split-Root System

**DOI:** 10.3389/fphys.2019.00786

**Published:** 2019-07-03

**Authors:** Nasir Iqbal, Sajad Hussain, Muhammad Ali Raza, Cai-Qiong Yang, Muhammad Ehsan Safdar, Marian Brestic, Ahsan Aziz, Muhammad Sikander Hayyat, Muhammad Ahsan Asghar, Xiao Chun Wang, Jing Zhang, Wenyu Yang, Jiang Liu

**Affiliations:** ^1^Key Laboratory of Crop Ecophysiology and Farming System in Southwest, Ministry of Agriculture, College of Agronomy, Sichuan Agricultural University, Chengdu, China; ^2^Department of Agronomy, College of Agriculture, University of Sargodha, Sargodha, Pakistan; ^3^Department of Plant Physiology, Slovak University of Agriculture, Nitra, Slovakia; ^4^Department of Botany and Plant Physiology, Faculty of Agrobiology, Food and Natural Resources, Czech University of Life Science Prague, Prague, Czechia; ^5^Institute of Ecological Agriculture, Sichuan Agricultural University, Chengdu, China

**Keywords:** enzymatic activity, chlorophyll fluorescence, polyethylene glycol, reactive oxygen species, Rubisco activity

## Abstract

Water deficiency significantly affects photosynthetic characteristics. However, there is little information about variations in antioxidant enzyme activities and photosynthetic characteristics of soybean under imbalanced water deficit conditions (WDC). We therefore investigated the changes in photosynthetic and chlorophyll fluorescence characteristics, total soluble protein, Rubisco activity (RA), and enzymatic activities of two soybean varieties subjected to four different types of imbalanced WDC under a split-root system. The results indicated that the response of both cultivars was significant for all the measured parameters and the degree of response differed between cultivars under imbalanced WDC. The maximum values of enzymatic activities (SOD, CAT, GR, APX, and POD), chlorophyll fluorescence (Fv/Fm, qP, ɸPSII, and ETR), proline, RA, and total soluble protein were obtained with a drought-tolerant cultivar (ND-12). Among imbalanced WDC, the enhanced net photosynthesis, transpiration, and stomatal conductance rates in T2 allowed the production of higher total soluble protein after 5 days of stress, which compensated for the negative effects of imbalanced WDC. Treatment T4 exhibited greater potential for proline accumulation than treatment T1 at 0, 1, 3, and 5 days after treatment, thus showing the severity of the water stress conditions. In addition, the chlorophyll fluorescence values of FvFm, ɸPSII, qP, and ETR decreased as the imbalanced WDC increased, with lower values noted under treatment T4. Soybean plants grown in imbalanced WDC (T2, T3, and T4) exhibited signs of oxidative stress such as decreased chlorophyll content. Nevertheless, soybean plants developed their antioxidative defense-mechanisms, including the accelerated activities of these enzymes. Comparatively, the leaves of soybean plants in T2 displayed lower antioxidative enzymes activities than the leaves of T4 plants showing that soybean plants experienced less WDC in T2 compared to in T4. We therefore suggest that appropriate soybean cultivars and T2 treatments could mitigate abiotic stresses under imbalanced WDC, especially in intercropping.

## Introduction

Soybean is an important crop throughout the world as a source of vegetable oil and protein. In southwest China, it is primarily intercropped with maize (Yan et al., 2010; Iqbal et al., 2018a). In maize-soybean planting, the height of the maize affects the microenvironment of the soybean in terms of light and moisture, having a negative effect on the soybean growth and development (Liu et al., 2017a). Earlier studies have shown imbalanced water deficit conditions for the soybean plants (Rahman et al., 2016). Interestingly, these imbalanced conditions significantly increased the production of biomass and yield and improved the quality of soybean (Iqbal et al., 2018b; Raza et al., 2018b). Reduced moisture leads to less photosynthesis, which reduces the dry matter production (Grassi and Magnani, 2005; Raza et al., 2018a). Therefore, the relative importance of physiological mechanisms should be recognized under imbalanced water deficit conditions.

Adequate water is needed for the development and growth of plants. The consequences of less than optimal water are oxidative stress and a reduction in photosynthetic characteristics (Guo et al., 2018). Reduction of photosynthesis results in decreased CO_2_ diffusion into the leaves because of lower internal (*g*_i_) and stomatal conductance (*g*_s_). It also results in the inhibition of photosynthesis due to limited leaf growth because of decreased cell proliferation (Lawlor and Tezara, 2009; Wu et al., 2018). More research is required to determine the activity and number of enzymes responsible for CO_2_ fixation and the regeneration of Rubisco-1,5-bisphosphate (RuBP). Rubisco (RuBP carboxylase or oxygenase) catalyzes the process of CO_2_ fixation (Susana et al., 2007) and is involved in the first phase of the Calvin Benson cycle. It accounts for 12–35% of leaf protein production in C_3_ plants (Evans and Seemann, 1989). Decreased RA may be involved in drought-associated photosynthetic rate (Flexas et al., 2006; Galmés et al., 2011). Imbalance in water deficit conditions may remarkably change the effect of Rubisco, but this has not been well elucidated yet.

Chlorophyll fluorescence measurements are an indicator of different drought responses of photosynthesis (Kalaji et al., 2018). Characteristics of chlorophyll fluorescence are a critical consideration as it is used to measure the quantum yield of photosystem II (PSII) and photoinactivation by determining the possible quantum yield under water limiting conditions (Batra et al., 2014). Photosynthesis is significantly affected by drought because it blocks the transport of energy from PSII to PSI (Siddique et al., 2016). It also leads to low chlorophyll fluorescence by reducing the palisade of spongy tissues and ultimate leaf thickness (Wang et al., 2018). In addition, the plant produces chemical signals in the dry portion of the root and this feed-forward mechanism reduces transpiration rate, stomatal opening, and shoot growth (Tardieu, 2016). These chemical signals are generally increased concentrations of abscisic acid (ABA) in the root that result in oxidative damage by unnecessary production of reactive oxygen species (ROS) (Beis and Patakas, 2015). ROS are regarded as second messengers in the ABA signaling pathway that regulate guard cell development (Yan et al., 2007). In the plasma membrane, the induction of hydrogen peroxide (H_2_O_2_) by ABA is an essential signaling event in modulating stomatal closure to decrease water loss through the activation of calcium-permeable channels (Pei et al., 2000). The presence of the plant defense system can protect the plant metabolism because ROS magnifies water stress leading to cell death by changing the properties of the cell membrane and causes oxidative damage to chlorophyll, protein, lipids, and DNA (Ahmad et al., 2010). Therefore, plants activate their antioxidant defense system to reduce the effects of ROS (Fan et al., 2017). Major enzymes which scavenge the ROS are peroxidase (POD), superoxide dismutase (SOD), ascorbate peroxidase (APX), glutathione reductase (GR), and catalase (CAT). Although the physiological impacts produced by water deficit are well documented, this remains a subject of high priority under imbalanced water deficit conditions.

By analyzing photosynthesis, it is possible to determine the degree of resistance to adverse conditions of the environment, e.g., excessive congestion (Prasad et al., 2015; Olechowicz et al., 2018). Photosynthesis is progressively reduced during drought, but the reason for this reduction is unclear at the seedling stage of soybean. Many studies propose the importance of diffusional limitations (stomatal and mesophyll) for most water deficit situations. Recently, there have been many investigations about the photosynthetic characteristics and antioxidant potential of soybean and other plants in water-limited conditions (Zivcak et al., 2013; Guo et al., 2018; Prasad et al., 2018), but these studies have focused primarily on photosynthetic gas exchange, chlorophyll fluorescence and antioxidant activities. However, it is unclear how the imbalanced water deficit influences photosynthesis, chlorophyll fluorescence and antioxidant activities of soybean seedlings. Therefore, this study aims to analyze the variations in antioxidant enzyme activity and photosynthetic characteristics of soybean under imbalanced water deficit conditions. The objectives of the current study were to (1) determine the antioxidant enzyme activities and ROS in terms of malondialdehyde (MDA) and H_2_O_2_, and SOD, CAT, GR, POD and APX, and (2) evaluate the photosynthetic characteristics, fluorescence parameters, total soluble protein, proline and Rubisco-activated enzyme.

## Materials and Methods

### Plant Growth Exposure and Experimental Conditions

**Figure 1:**
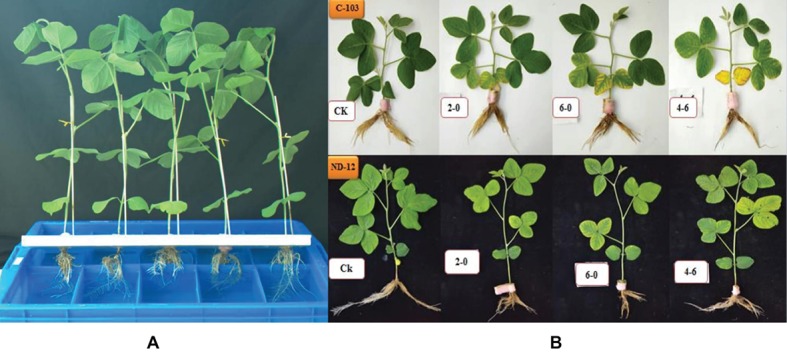
**(A)** Schematic representation of equally divided soybean roots in the solution box. **(B)** Phenotypic appearance of soybean cultivars after 5 days of PEG treatments; C-103 with a white background and ND-12 with a black background.

In this experiment, two soybean cultivars, ND-12 (drought-tolerant) and C-103 (drought-susceptible) were used in the greenhouse of Sichuan Agricultural University, Chengdu, China (29° 59′N, 103° 00′E). Seven-day-old seedlings (at the VC stage) were transplanted to plastic boxes containing half-strength Hoagland solution. Plant growth conditions were normal in the greenhouse, maintaining a 12 h photoperiod, 24/20°C day/night temperature and approximately 60–70% relative humidity. The photosynthetically active radiation was 279 μmol m^−2^ s^−1^. At the V_3_ stage, healthy plants were selected and their roots were equally divided in the solution boxes (Figure 1). Horizontal foam (polyurethane) was used to hold the soybean plants, which were kept under normal conditions for 1 week before exposure to stress treatments.

At the V_4_ stage, combinations of four different treatments were imposed: 0% polyethylene glycol (PEG) on both sides as control (T1 = 0%: 0%), 2% PEG on side A and 0% PEG on side B (T2 = 2%A: 0%B), 6% PEG on side A and 0% PEG on side B (T3 = 6%A: 0%B), and 4% PEG on side A and 6% PEG on side B (T4 = 4%A: 6%B). PEG-6000 was used to produce osmotic potential. Each treatment had three boxes each having five plants and had internal and external sizes of 530 mm × 350 mm × 130 mm and 590 mm × 380 mm × 140 mm, respectively. Leaf samples of each treatment were harvested in triplicate. Samples to be used for analysis were frozen in liquid nitrogen and stored with their tags at −80°C.

### Enzymatic Activities Measurement

All ROS and enzymatic activities were measured from one of the most recently expanded trifoliate leaves. Leaf samples were collected at 0, 1, 3, and 5 days and stored at −80°C for analysis. Commercial kits were used to determine the total soluble protein, superoxide dismutase (SOD), glutathione reductase (GR), peroxidase (POD), ascorbate peroxidase (APX), catalase (CAT), malondialdehyde (MDA), and hydrogen peroxide (H_2_O_2_) as per the manufacturer’s instructions (Supplementary data). These commercial kits were ordered from Nanjing Jiancheng Bioengineering Institute, Nanjing, China.

### Proline Measurement

Free proline was measured according to a previously published method (Bates et al., 1973). Soybean leaves were collected at reproductive stage R5 and were freeze-dried and extracted with a 5 ml extraction solution of 3% sulfosalicylic acid. Then, 2 ml supernatant was reacted with glacial acid (2 ml) and acid ninhydrin (3 ml); the solution was boiled for 40 min. After cooling the samples at room temperature, 5 ml toluene was added and mixed by vortexing. The spectrophotometer (Mapada-V-1100D) read the absorbance at 520 nm.

### Photosynthetic Characteristics and Chlorophyll Content

We used a portable photosynthesis system (*Model LI-6400, LI-COR Inc.,* Lincoln, NE) to determine the net photosynthetic rate (*P*_N_), stomatal conductance (*g*_s_) and transpiration (*E*). Photosynthetic parameters were measured through the latest, fully expanded leaves between 08:00 to 11:00 h. The following settings of PARi = 1,000, flow = 500 μmol mol^−1^, stomatal ratio = 0.5, and reference CO_2_ concentration = 400 μmol mol^−1^ were used. Leaf chlorophyll contents were noted with the help of the SPAD-502 (*Minolta*, Japan) apparatus.

### Chlorophyll Fluorescence Parameters

In this experiment, Fluor Technologia software (*Fluor Images*, United Kingdom) measured the chlorophyll fluorescence. We used plastic bags to preserve fully expanded leaf samples and placed them in an icebox covered with a lid to prevent the entry of direct light. Later, the samples were passed to a fluorescence analyzing device by using software. By placing them under dark and light conditions for 20 min., their photochemical efficiency (ɸPSII), photochemical quenching (qP) and electron transport rate (ETR) were determined by the FluorImager software, Technologia LTD (version 2.2.2.2) (Pan et al., 2017).

### Analysis of Plant Rubisco-Activated Enzyme

The Rubisco ELISA kit (96 micropores) was purchased from Shanghai Fu Life Industry Co. Ltd., Shanghai, China. To measure the Rubisco-activated enzyme, 1 g of frozen leaf samples were ground with the help of a mortar and pestle and an icebox, using 2 ml of 50 mmol L^−1^ phosphate buffer solution (pH 7.8). The solution was centrifuged at 7000 rcf at 4°C for 15 min. The level of plant Rubisco activase was determined by the double antibody sandwich method. The micropore plate encapsulated the Rubisco activase antibody to form a solid phase antibody. This was added to the micropore of the monoclonal antibody. The 40 μl of phosphate buffer solution as a sample diluent was added first, followed by 10 ml of the sample solution in the micropore plate. A plastic film sealed the micropore plate and kept it incubated at 37°C for 30 min. This incubation was repeated five times. The 3,3′5,5′-tetramethylbenzidine was transferred under the catalysis of horseradish peroxidase enzyme, which first turned blue and finally to a yellow color under the action of an acid. The absorbance was measured after adding the stop solution within 15 min at a 450 nm wavelength by an enzyme marker. A standard curve was used to calculate the sample and the RA was expressed as U/g (Hussain et al., 2019).

### Statistical Analysis

The experimental data analysis used Statistics software (Statistics 8.1. Tallahassee, FL, USA), while the figures were drawn using Microsoft Office 2010. Duncan’s multiple range tests compared the treatment means, with statistically significant differences at *p* ≤ 0.05.

## Results

### Effect of Imbalanced Water Deficit on Soybean ROS

**Figure 2:**
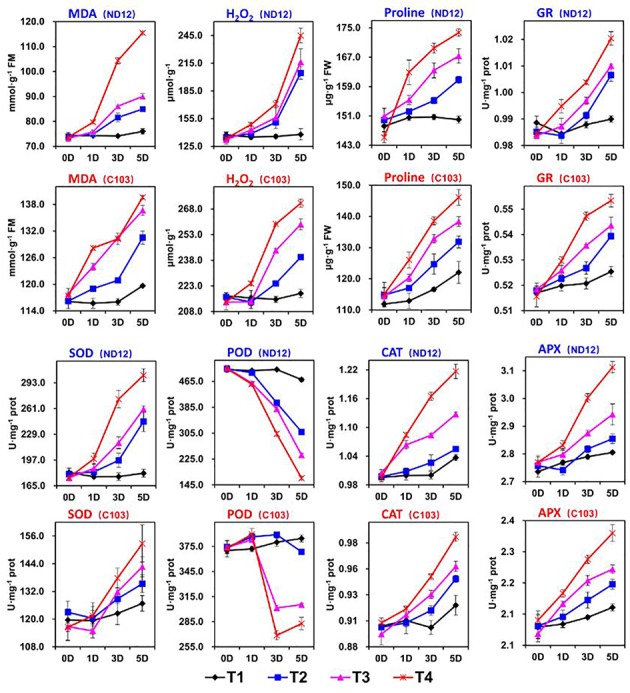
Effect of different split-root PEG treatments on reactive oxygen species, enzymatic activity and proline of two soybean cultivars, ND-12 (drought-resistant) and C-103 (drought-susceptible). (Mean ± SE). T1 (0%: 0%), T2 (2%A: 0%B), T3 (6%A: 0%B), and T4 (4%A: 6%B).

In this experiment, we examined the effects of imbalanced water deficit on the levels of MDA and H_2_O_2_ in the leaves of soybean seedlings. Different split-root PEG treatments (SRP) indicated a significant (*p* < 0.05) effect on the ROS levels in ND-12 and C-103 from 0 to 5 days (Figure 2). Between both cultivars, the highest average MDA (131.6 nmol mg^−1^ prot) and H_2_O_2_ (247.1 μmol g^−1^) were measured in C-103, while the lowest MDA (91.63 nmol mg^−1^ prot) and H_2_O_2_ (200.8 μmol g^−1^) were recorded in ND-12 at the 5^th^ day of sampling. All the SRP treatments significantly affected the ROS levels in soybean seedlings; the maximum (127.5 nmol mg^−1^ prot and 258.1 μmol g^−1^) and minimum (97.8 nmol mg^−1^ prot and 178.2 μmol g^−1^) values of MDA and H_2_O_2_ were measured in the T4 and T1 treatments, respectively, on the 5^th^ day of sampling (Figure 2). The interactive effect of soybean cultivars and SRP treatments for MDA was significant for all sampling days except at day 0. On average, on the 5^th^ day of sampling, T4 increased the levels of MDA and H_2_O_2_ by 30 and 45%, respectively, compared to T1 (Figure 2).

### Effect of Imbalanced Water Deficit on Soybean Antioxidative Enzymes

Plants activate their defense system to eliminate ROS. Major enzymes that scavenge ROS include CAT, SOD, APX, and POD. In this experiment, SRP treatments and soybean cultivars showed a significant effect on the activities of SOD, CAT, GR, APX, and POD, and maximum activities of these enzymes were obtained by ND-12 and C-103 (Figure 2). Furthermore, enzymatic activities of SOD, GR, CAT, and APX increased with the increase in SRP concentration, with their maximum activities measured in T4 and minimum activities in treatment T1 on the 5^th^ day of measurement. Overall, on the 5^th^ day of measurement, treatment T4 increased the SOD, GR, CAT, and APX activities by 48, 4, 13, and 10%, respectively, over treatment T1. However, the activity of POD significantly (*p* < 0.05) decreased with the increase in PEG concentration. Specifically, on the 5^th^ day of measurement, the activity of POD reduced by 91, 58, and 26% in T4, T3, and T2, respectively, compared to that in T1. The interactive effect of SRP treatments and soybean cultivars for GR was found nonsignificant and significant at 0, 1, and 5 day and at 3 day intervals, respectively; for POD and CAT, it was found nonsignificant and significant at 0 days and at 1, 3, and 5 days, respectively; and for SOD and APX, it was found nonsignificant and significant at 0 and 1 days and at 3 and 5 days, respectively (Figure 2).

### Effect of Imbalanced Water Deficit on Proline

Plants accumulate free proline under water-limited conditions. In this study, cultivars exhibited significant differences in free proline. The highest (162.9 μg g^−1^) and the lowest (134.5 μg g^−1^) proline accumulation in soybean seedlings were noted in cultivar ND-12 and C-103, respectively, on the 5^th^ day of measurement. Additionally, free proline significantly increased with SRP concentration. The maximum (159.9 μg g^−1^) and minimum (136.0 μg g^−1^) values of free proline were measured in the T4 and T1 treatments, respectively, on the 5^th^ day of sampling (Figure 2). The interactive effect of SRP treatments and soybean cultivars for the accumulation of free proline was nonsignificant.

### Effect of Imbalanced Water Deficit on Photosynthetic Parameters

**Figure 3:**
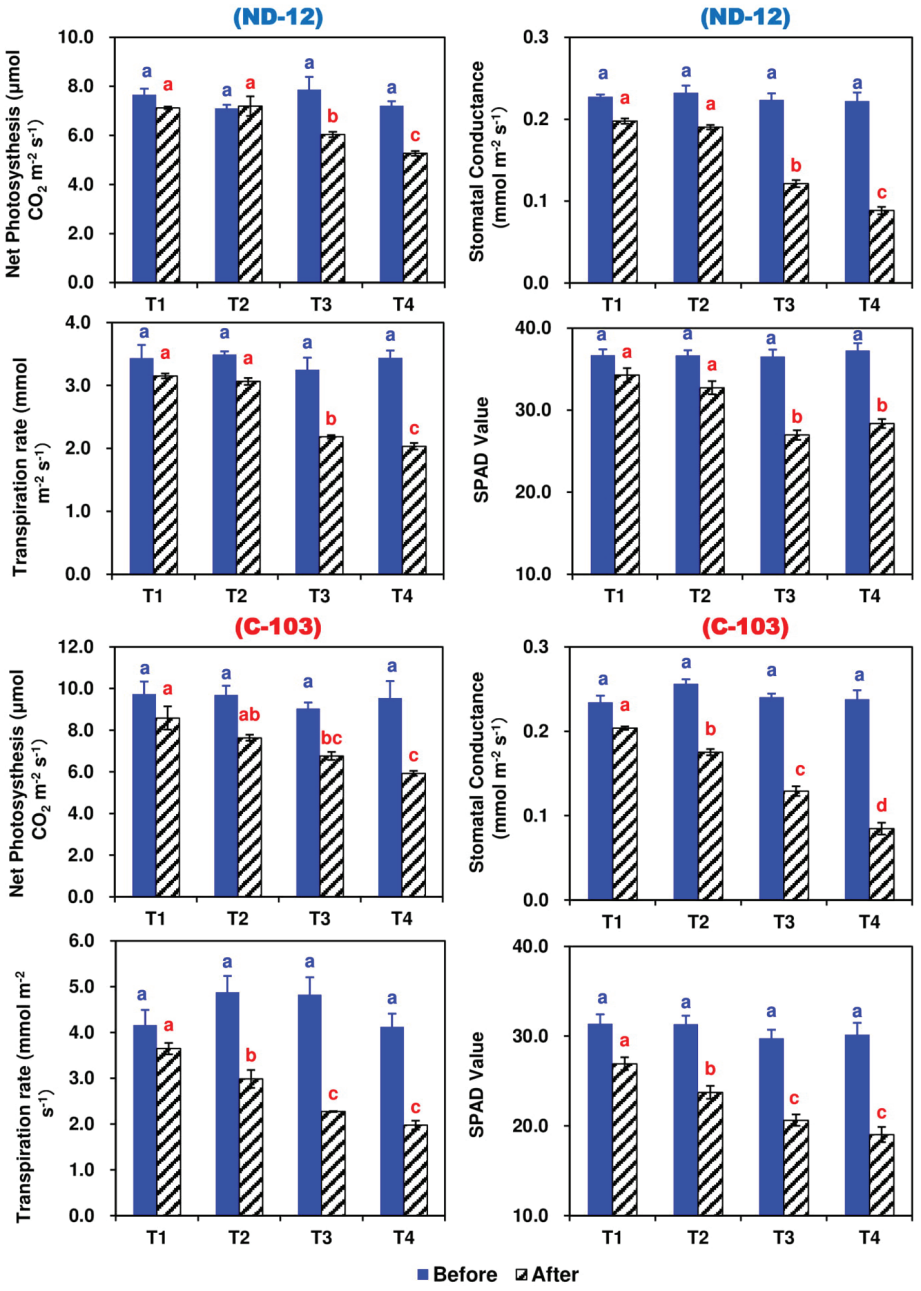
Effect of different split-root PEG treatments on photosynthetic parameters of two soybean cultivars, ND-12 (drought resistant) and C-103 (drought susceptible). (Mean ± SE), same small letters are not significantly different at *p* < 0.05. T1 (0%: 0%), T2 (2%A: 0%B), T3 (6%A: 0%B), and T4 (4%A: 6%B).

The effect of different SRP treatments and soybean cultivars on the photosynthetic rate (*P*_N_), transpiration rate (*E*), stomatal conductance (*g*_s_), and chlorophyll content (*Chl*) in the leaves of soybean are shown in Figure 3. Prior to the applied stress, the values of these traits were significantly higher among all treatments and no changes were observed. However, after 5 days of stress, different SRP treatments significantly affected the *P*_N_, *E*, *g*_s_, and Chl contents of the soybean cultivars. Maximums for *P*_N_ (7.8545 and 7.4134 CO_2_ m^−2^ s^−1^), *E* (3.3980 and 3.0253 mmol m^−2^ s^−1^), *g*_s_ (0.2007 and 0.1828 mmol m^−2^ s^−1^), and Chl contents (30.60 and 28.23) were measured in T1 and T2, while minimums for *P*_N_ (5.6004 CO_2_ m^−2^ s^−1^), *E* (2.0056 mmol m^−2^ s^−1^), *g*_s_ (0.0866 mmol m^−2^ s^−1^), and Chl contents (23.70) were noted under the T4 treatment. Among soybean cultivars, the highest values of *P*_N_ (7.2273 CO_2_ m^−2^ s^−1^) and *E* (2.7206 mmol m^−2^ s^−1^) were noted for C-103, whereas the highest values of *g*_s_ (0.1495 mmol m^−2^ s^−1^) and Chl contents (30.58) were observed in cultivar ND-12. The interactive effect of SRP treatments and soybean cultivars for photosynthetic parameters were found to be significant (Figure 3).

### Effect of Imbalanced Water Deficit on Chlorophyll Fluorescence Parameters of Soybean

**Figure 4:**
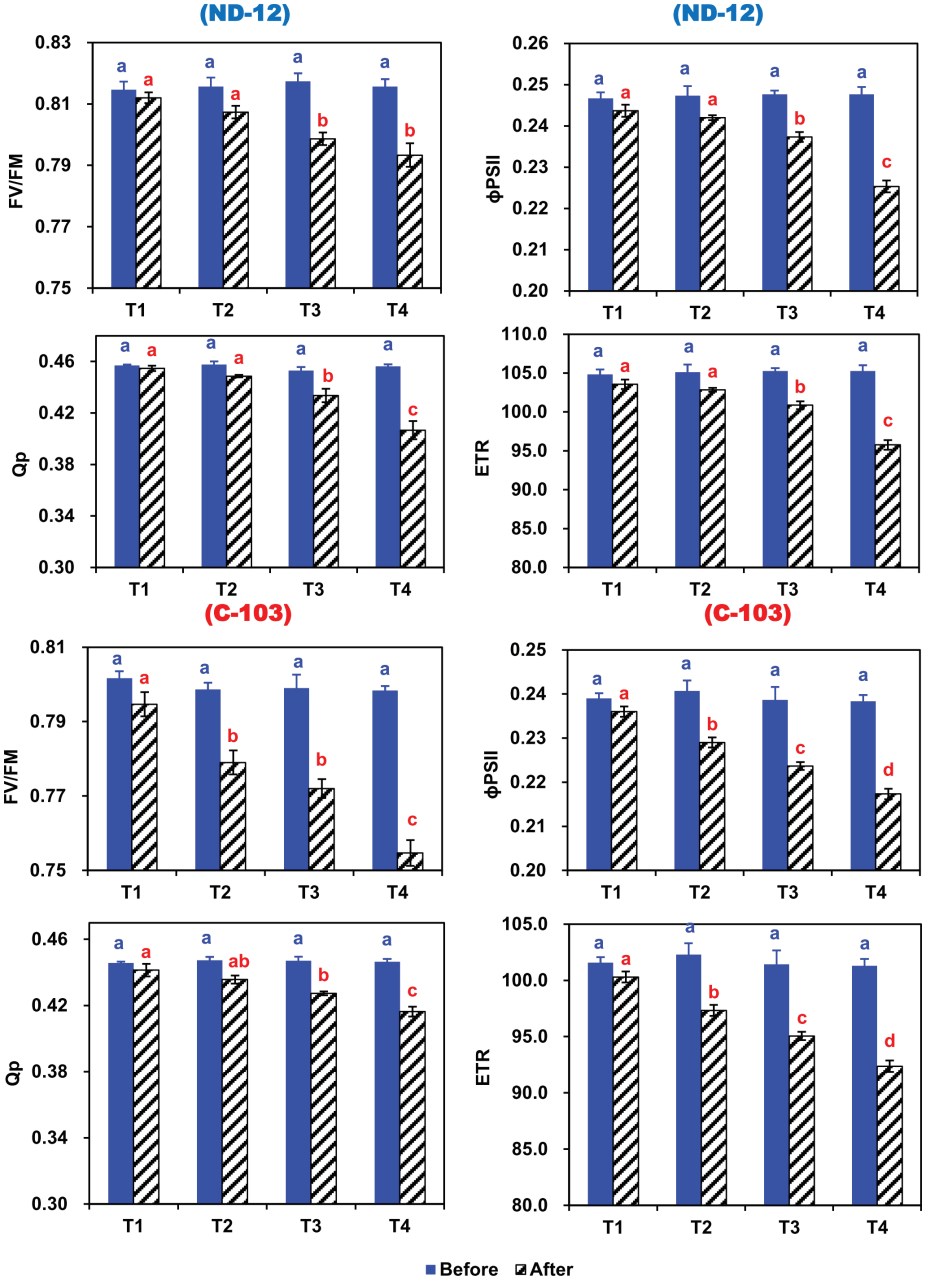
Effect of different split-root PEG treatments on chlorophyll fluorescence parameters of two soybean cultivars ND-12 (drought-resistant) and C-103 (drought-susceptible). (Mean ± SE), same small letters are not significantly different at *p* < 0.05. T1 (0%: 0%), T2 (2%A: 0%B), T3 (6%A: 0%B), and T4 (4%A: 6%B). Maximum quantum yield (Fv/Fm), effective quantum yield of photosystem (ɸPSII), photochemical quenching (qP), and electron transport rate (ETR).

In this experiment, the chlorophyll fluorescence significantly changed during the experimental period in response to induced imbalanced water deficit conditions (Figure 4). Prior to the applied stress, there was a nonsignificant difference in maximum quantum yield (Fv/Fm), photochemical quenching (qP), effective quantum yield of photosystem (ɸPSII) and electron transport rate (ETR). After 5 days of stress, the Fv/Fm, qP, ɸPSII, and ETR of both soybean cultivars showed significant changes under different SRP treatments. In soybean cultivars, the maximum (0.8028, 0.4359, 0.2371, and 100.76) and minimum (0.7751, 0.4302, 0.2265, and 96.26) values of Fv/Fm, qP, ɸPSII, and ETR were observed in ND-12 and C-103, respectively. Among SRP treatments, the maximum values of Fv/Fm (0.8033 and 0.7932), qP (0.4480 and 0.4422), ɸPSII (0.2398 and 0.2355), and ETR (101.93 and 100.09) were noticed under treatments T1 and T2, while minimum concentrations of Fv/Fm (0.7740), qP (0.4115), ɸPSII (0.2213), and ETR (94.07) were measured in T4. The interactive effect of SRP treatments and soybean cultivars for Fv/Fm, qP, ɸPSII, and ETR were found to be significant. Overall, relative to treatment T1, the Fv/Fm, qP, ɸPSII, and ETR decreased by 4, 9, 8, and 8%, respectively, under the T4 treatment, indicating that changes in photosynthetic rate under imbalanced water deficit conditions were directly associated with the changes in chlorophyll fluorescence parameters.

### Effect of Imbalanced Water Deficit on Rubisco Activity (RA) and Total Soluble Protein of Soybean

**Figure 5:**
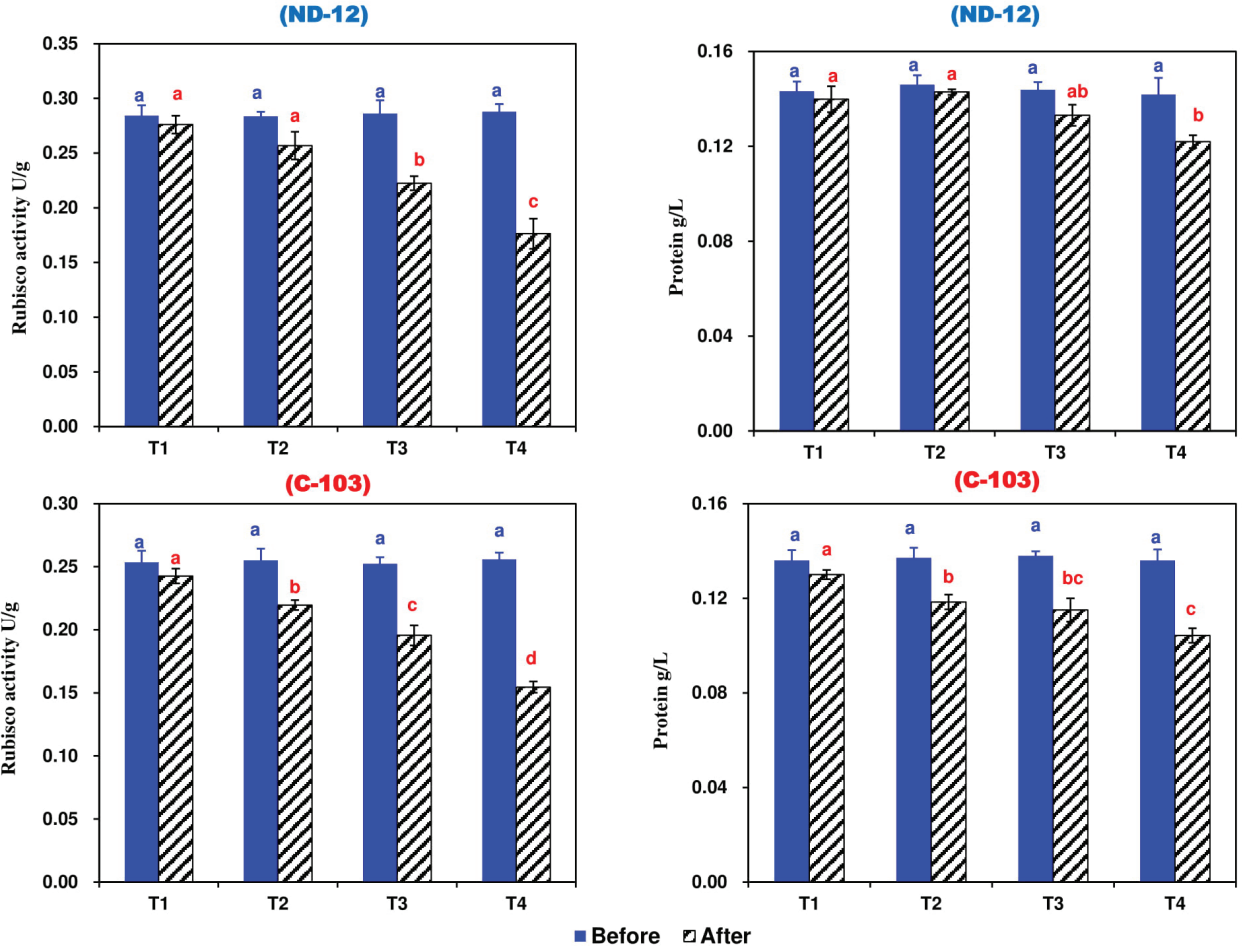
Effect of different split-root PEG treatments on Rubisco activity and total soluble protein of two soybean cultivars, ND-12 (drought-resistant) and C-103 (drought-susceptible). (Mean ± SE), same small letters are not significantly different at *p* < 0.05. T1 (0%: 0%), T2 (2%A: 0%B), T3 (6%A: 0%B), and T4 (4%A: 6%B).

Prior to the stress applied, the RA and total soluble protein were significantly higher among all treatments compared to the control and no changes were observed, while after stress treatment, SRP treatments significantly affected the RA and total soluble protein of soybean cultivars. The maximum RA (0.2329 U g^−1^) and total soluble protein (0.1345 g L^−1^) were noted in ND-12, while the minimum RA (0.2031 U g^−1^) and total soluble protein (0.1169 g L^−1^) were observed in C-103. Among the SRP treatments, the maximum RA (0.2593 and 0.2382 U g^−1^) and total soluble protein (0.1349 and 0.1307 g L^−1^) were measured in T1 and T2, while the minimum RA (0.1654 U g^−1^) and total soluble protein (0.1131 g L^−1^) were noted under the T4 treatment. The interactive effect of different SRP treatments and soybean cultivars for RA and total soluble protein were found to be significant (Figure 5). These results suggest that the RA and total soluble protein were inhibited more obviously in ND-12 than in C-103, which in turn improves the chlorophyll fluorescence and photosynthetic parameters of ND-12 under the imbalanced water deficit conditions.

### Correlation

**Figure 6:**
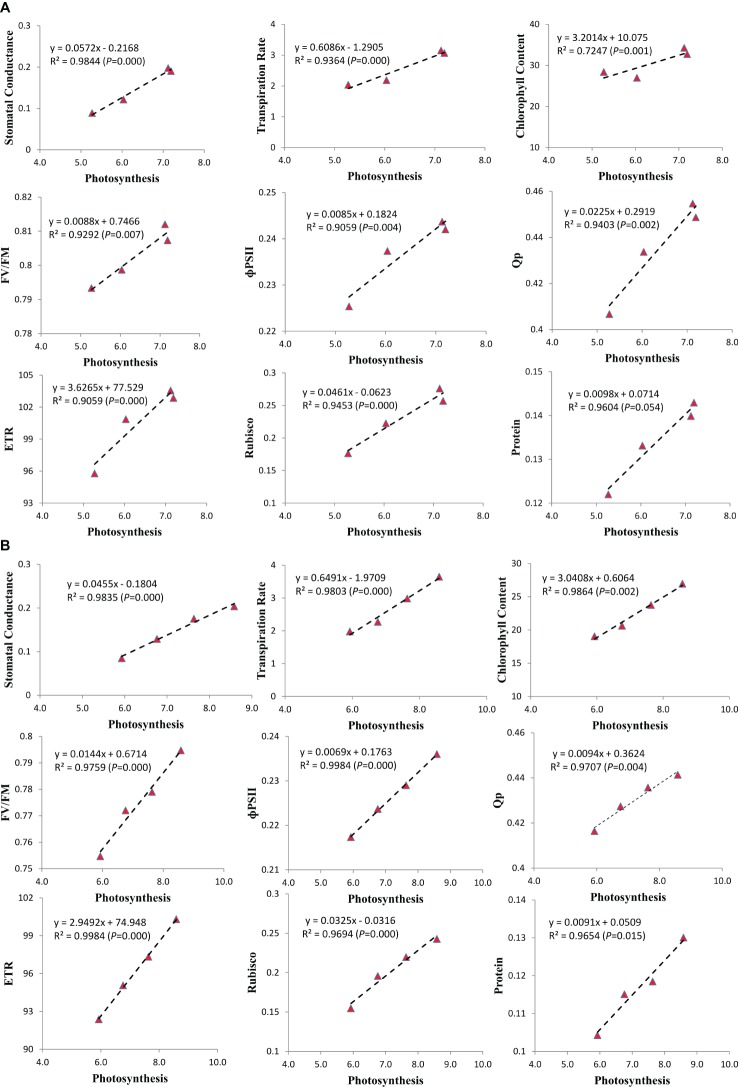
Relationship of photosynthesis with the stomatal conductance, transpiration rate, chlorophyll content, chlorophyll fluorescence parameters, Rubisco activity and protein of soybean seedlings in **(A)** (ND-12) and **(B)** (C-103). Correlation coefficients (R) are calculated and significance (P) represents significance at the 0.05 probability.

To recognize the most critical photosynthetic parameters affecting soybean growth, the relationship between the increasing photosynthetic rate and photosynthetic characteristics was drawn (Figure 6). Among the photosynthetic parameters of soybean cultivars, the stomatal conductance, transpiration rate, chlorophyll content, chlorophyll fluorescence characteristics (FV/FM, PSII, qP, and ETR), RA, and protein content of both cultivars increased with the increase in photosynthetic rate. We found that the stomatal conductance (*R*^2^ = 0.9844 and 0.9835, *p* = 0.000 and 0.000), transpiration rate (*R*^2^ = 0.9364 and 0.9803, *p* = 0.000 and 0.000), chlorophyll content (*R*^2^ = 0.7247 and 0.9864, *p* = 0.001 and 0.002), FV/FM (*R*^2^ = 0.9292 and 0.9759, *p* = 0.007 and 0.000), ɸPSII (*R*^2^ = 0.9059 and 0.9984, *p* = 0.004 and 0.000), Qp (*R*^2^ = 0.9403 and 0.9707, *p* = 0.002 and 0.004), ETR (*R*^2^ = 0.9059 and 0.9984, *p* = 0.000 and 0.000), RA (*R*^2^ = 0.9453 and 0.9694, *p* = 0.000 and 0.000) and protein content (*R*^2^ = 0.9604 and 0.9654, *p* = 0.054 and 0.015) of ND-12 and C-103, respectively, at the V4 soybean growth stage were strongly and positively (*p* < 0.05) related to the increasing photosynthetic rate of soybean plants. The correlation coefficient between all the measured parameters and increasing photosynthetic rate for the mean data sets of cultivars ND-12 and C-103 were all higher than 0.00 (*p* < 0.05).

## Discussion

### Effect of Imbalanced Water Deficit on Soybean ROS and Antioxidant Enzymes

Environmental stresses have been known to cause oxidative injuries by increasing the levels of ROS; excessive production of ROS can severely damage plant metabolism (Sinha et al., 2016). ROS enhances the effects of water stress by disturbing the cell membrane of plants and causing oxidative impairment to chlorophyll pigments, lipids, protein, and DNA, which altogether lead to cell death. In this study, a clear increase in MDA and H_2_O_2_ content was recorded in both cultivars in response to enhanced split-root PEG stress. However, better protection from oxidative damage was observed in the ND-12 cultivar than in C-103 due to its lowered MDA and H_2_O_2_ content. The highest concentration of H_2_O_2_ is attributed to lipids, proteins, and nucleic acid oxidization, which led to inactivation of photosystems I and II. These findings are consistent with the previous reports where MDA and H_2_O_2_ content increased in soybean leaves under water-limited conditions (Türkan et al., 2005; Shen et al., 2010).

To prevent cellular damage, plants mobilize the antioxidant defense system to eliminate ROS. In this study, soybean cultivars increased enzyme activities, which may be attributed to the protection of plants from an imbalanced water deficit. These findings are consistent with an earlier study in which water deficit caused an increase in the enzymatic activities except for POD in soybean (Shen et al., 2015). In addition, enzyme activities varied between the two soybean cultivars. However, these changes in enzymatic activities are dependent on plant age, species, treatment durations, and experimental conditions (Pan et al., 2006). Higher values of ROS and lower antioxidant enzyme activities in C-103 might be due to its low drought tolerance. Eventually, these conditions caused injuries to the plant by increasing ROS production.

The increase in proline is a common response of plants under water deficit conditions. Proline protects the stressed cells by adjusting intercellular osmotic potential in soybean (Heerden and Kruger, 2002). In this study, soybean cultivars increased the free proline content, which may be attributed to high water retention. A higher value of proline accumulation in ND-12 showed its high water retention ability compared to C-103. The results of this study are in agreement with those of earlier investigators, who noted a significant increase in free proline in soybean in response to water stress (Shen et al., 2010; Sharma et al., 2012).

### Effect of Imbalanced Water Deficit on Photosynthetic Characteristics of Soybean

Split-root drought is a smart approach to minimize water loss and maximize crop productivity (Iqbal et al., 2019). Plants have the ability to perceive dried soil and reduce water use by regulating certain physiological and biochemical changes in the dry segment of the root zone (Mingo et al., 2004). In the present studies, two soybean cultivars were grown in Hoagland solution to know the physiological response of soybean seedlings against imbalanced water deficit conditions. *P*_N_, *g*_s_, and *E* were significantly higher before the treatment application. Plants under SRP treatments maintained significantly lower values of these parameters. Retardation of photosynthesis resulted in low agricultural productivity, and a decrease in *P*_N_ was attributed to the decrease in *g*_s_ and intercellular CO_2_ concentration in drought-stressed plants (Chaves et al., 2009). These results are consistent with the earlier report where *P*_N_, *g*_s_, and *E* were significantly decreased in partial-root drying of tomato grown in a greenhouse (Campos et al., 2009). However, these parameters varied between both cultivars. In ND-12, the values of *P*_N_, *g*_s_, and *E* were not significantly different under treatments T1 and T2, which was the reason for its high drought resistance.

A sharp decrease in RA is considered an early response to drought stress in soybean (Bota et al., 2004). This decrease was significantly higher in drought-stressed plants compared to the control. In this study, the results showed that RA remained higher before treatment application and a strong reduction was often found after T2 and T3 in ND-12 and C-103, respectively. Treatment T2 reduced RA by 10% in C-103 and only 7% in ND-12. As described above, *P*_N_ and *g*_s_ were more inhibited in ND-12 than in C-103 under imbalanced water deficit treatments. These results are in line with earlier findings that the downregulation of RA is induced by *g*_s_ (Flexas et al., 2006) and suppression of Rubisco could be the possible reason for the low photosynthetic rate (Pietrini et al., 2003). However, the mechanism for decreased RA seems species-dependent. In principle, decreased RA could be the consequence of decreased soluble protein concentration (Bota et al., 2004). In this study, RA and total soluble protein were more inhibited in ND-12 than in C-103 under imbalanced water deficit treatments. However, it cannot be evaluated in terms of alterations in total soluble protein contents, as there are some other possibilities (Zhang et al., 2016; Liu et al., 2017b). Therefore, the physiological meaning of these adjustments deserves detailed attention in the future.

In addition, the chlorophyll content decreased as the levels of PEG increased in both cultivars. Relatively lower values of chlorophyll were found for C-103, which supports the view that this cultivar is more affected by imbalanced water deficit conditions. Reduction in chlorophyll content is attributed as a typical symptom of oxidative stress and has been reported in earlier studies (Gunes et al., 2008; Masoumi et al., 2010). Chlorophyll degradation and pigment photo-oxidation caused by chlorophyll reduction ultimately inactivates photosynthesis (Hajihashemi and Ehsanpour, 2013). Therefore, the current study clearly showed that the reduction of chlorophyll content also affected soybean photosynthesis.

Increased photosynthetic capacity accompanies a high quantity of electrons passing through PSII (Yao et al., 2017). Under different environmental conditions (sensitivity and convenience), parameters derived from chlorophyll fluorescence measurements can indicate changes in photosynthesis (Dai et al., 2009). A decrease in plant growth under drought is due to lower energy absorbed by the leaf and subsequently translocated to PSII (Rahbarian et al., 2011; Kalaji et al., 2014). In the present study in soybean plants, as the stress increased, Fv/Fm, qP, PSII, and ETR were significantly lower. However, the decrease in chlorophyll fluorescence parameters in ND-12 occurred later than that in C-103. The results showed that the limitations of these parameters were similar to that of *P*_N_. However, no significant decrease in chlorophyll fluorescence parameters was observed in ND-12 (drought-resistant) under T1 and T2, suggesting that the PSII structural integrity of the resistant soybean cultivar was not injured by imbalanced water deficit conditions. Our results are similar to those of Piper et al. (2007) and Mao et al. (2018), where the Fv/Fm ratio was higher in resistant than in susceptible cultivars (Piper et al., 2007; Mao et al., 2018). Thus, our results suggest that under imbalanced water deficits conditions, the efficiency of PSII increases in the resistant cultivar. Improving the energy transport from PSII to PSI may enhance photosynthesis.

## Conclusion

This research will ensure a better understanding of the response mechanisms of plants to imbalanced WDC. Biased application of PEG treatments reduced the oxidative stress by upregulating the enzymatic activities of key enzymes (SOD, POD, CAT, GR, and APX). Compared to normal conditions, the split-root PEG treatment T4 increased the MDA and H_2_O_2_ of soybean plants by 30 and 45%, respectively, on the 5^th^ day of sampling. In response to ROS, antioxidant activities of SOD, CAT, GR, and APX improved by 48 13, 4, and 10%, respectively, in T4 over T1 on the 5^th^ day of measurement in both cultivars. Furthermore, imbalanced WDC (T4) decreased the efficiency of PSII by regulating chlorophyll fluorescence (Fv/Fm, qP, PSII, and ETR) by 2, 12, 8, and 8% in ND-12 and 5, 6, 9, and 9% in C-103, respectively. Additionally, the activities of Rubisco were significantly reduced under T4 treatment, which in turn decreased the photosynthesis of soybean seedling. Furthermore, increased enzymatic activity, photosynthetic efficiency, RA and total soluble protein upon high PEG application in both cultivars ensured healthier plant growth, particularly in the drought-tolerant cultivar (ND-12). The results of the current study suggested that the appropriate cultivars and imbalanced WDC of T2 can modify the photosynthetic performance of plants, especially in intercropping systems.

## Author Contributions

NI and C-QY helped in data curation. SH and MR helped in formal analysis. WY, WC, and ZJ helped in funding acquisition. NI and SH developed the methodology. JL collected the resources. WY and JL helped in supervision MS and MH helped in validation. NI, SH, and MB helped in writing—original draft. MS, AA, and MA helped in writing—review and editing.

### Conflict of Interest Statement

The authors declare that the research was conducted in the absence of any commercial or financial relationships that could be construed as a potential conflict of interest.
