# Supplementary material for: Drought Tolerance of Soybean (Glycine max L. Merr.) by Improved Photosynthetic Characteristics and an Efficient Antioxidant Enzyme Activities Under a Split-Root System
Source: Front Physiol. 2019 Jul 3;10:786. doi: 10.3389/fphys.2019.00786 (PMC6621490; doi:10.3389/fphys.2019.00786)
Supplement: Supplementary file 1 [file Data_Sheet_1.pdf]

Supplementary data

Malondialdehyde (MDA)

A commercial kit (TBA Method, Serial No. A003) was used to determine the MDA content. Fresh leaves (0.1 g) were homogenized with reagent 1 (0.1 ml). Samples were mixed sufficiently by shaking the test tube stand. Reagents 2 (3 ml) and 3 (1 ml) were added and mixed sufficiently by vortexing. Samples were placed in a 95℃ water bath for 40 min. After cooling at room temperature, they were centrifuged at 4000 rpm for 10 min. The supernatant was taken and absorbance of all tubes was measured at 532 nm in the spectrophotometer.

Hydrogen peroxide (H_2O_2)

A commercial kit (A064-1) was used to determine the H_2O_2. A total of 0.5 g of fresh leaves was homogenized with reagents 1 and 2 (1 ml). The mixture was blended and regulated by the spectrophotometer with double distilled water. Optical density (OD) was recorded at 405 nm.

Superoxide dismutase (SOD)

A commercial kit (A001-1) was ordered and the manufacturer's instructions were followed to measure the SOD. A total of 0.1 g leaf samples was grounded in 5 ml phosphate buffer solution (0.1 mol/L pH 7.4). After grinding, we centrifuged them at 4000 rpm for 10 min. Supernatant was taken and mixed with reagents 1 (1 ml), 2 (0.1 ml), 3 (0.1 ml) and 4 (0.1 ml). Samples were mixed sufficiently by vortex and placed in a 37℃ thermostatic water bath for 40 min. After cooling at room temperature, a chromogenic agent (2 ml) was added into each sample. They were mixed sufficiently and placed at room temperature for 10 min. OD values of all samples were measured at 550 nm in the spectrophotometer.

Peroxidase (POD)

A commercial kit (A084-3) was used to determine the POD. A total of 0.1 g leaf samples was grounded in 5 ml phosphate buffer solution (0.1 mol/L pH 7.4). After grinding, we centrifuged them at 3500 rpm for 10 min. The supernatant was mixed with reagent I (2.4 ml), reagent II solution (0.3 ml) and reagent III standard solution (0.2 ml). Samples were kept in a water bath at 37°C for 30 min. After adding reagent IV (1 ml), the mixture was mixed thoroughly and centrifuged at 3,500 rpm for 10 min. The supernatant was extracted and the OD value was measured at 420 nm.

Catalase (CAT)

A commercial kit (A007-2) was ordered, and the visible light method was used to measure the CAT. Fresh leaves (0.1 g) were homogenized with 0.9 ml distilled water. Samples were centrifuged at 2500rpm for 10 min. The supernatant (0.05 ml) was taken and mixed sufficiently with reagents 1 (1 ml) and 2 (0.1 ml). Samples were reacted at 37℃ for exactly 1 min. After that, reagents 3 (1 ml) and 4 (0.1 ml) were added and the OD value of each sample was measured at 405 nm.

Ascorbate peroxidase (APX)

APX activity was determined by measuring the consumption of ascorbate by following absorbance at 290 nm. One unit of APX activity was defined as the amount of enzyme required to consume 1 µmole ascorbate min^{-1}.

Glutathione Reductase (GR)

The Assay Kit (A062) was ordered and GR activity was determined by measuring the enzyme-dependent oxidation of NADPH by following absorbance at 340 nm. One unit of GR activity was defined as the amount of enzyme that oxidized 1 μmole NADPH min^{-1}.

Total protein analysis.

A commercial kit (A045-2) was ordered and the Coomassie Brilliant Blue method was used to measure the total protein. The stock solution was diluted with distilled water at a ratio of 1:4 (5 times dilution) to prepare a working solution. A total of 0.1 g leaf samples was homogenized in 0.9 ml phosphate buffer solution (pH 7.4). Samples were centrifuged at 3000 rpm for 10 min. The supernatant was taken and diluted with physiological saline at a ratio of 1:9 to prepare 1% tissue homogenate. A total of 0.05 ml sample was mixed with 3 ml working solution and placed quiescently for 10 min. OD values of all tubes were measured at 595 nm.
